# Oxidative stress in dogs with chronic inflammatory enteropathy treated with allogeneic mesenchymal stem cells

**DOI:** 10.1007/s11259-023-10265-0

**Published:** 2023-11-28

**Authors:** José Ignacio Cristóbal, Francisco Javier Duque, Jesús Usón-Casaús, María Salomé Martínez, María Prado Míguez, Eva María Pérez-Merino

**Affiliations:** 1 Departamento de Medicina Animal, Unidad de Cirugía, Facultad de Veterinaria, Universidad de Extremadura, Veterinaria UEx. Avenida de La Universidad S/N, 10003 Cáceres, Spain; 2 Unidad de Toxicología, Facultad de Veterinaria, Universidad de Extremadura, 10003 Cáceres, Spain

**Keywords:** Chronic inflammatory enteropathy, Mesenchymal stem cells, Malondialdehyde, Glutathione, Albumin

## Abstract

The search for new biomarkers in patients with chronic inflammatory enteropathy (CIE) is ongoing in the human and veterinary medicine fields. Oxidative stress biomarkers (malondialdehyde [MDA], reduced glutathione [GSH], and albumin) have been studied in humans with chronic enteropathies, but among them, only albumin has been studied in dogs with CIE. Moreover, the effect of mesenchymal stem cell (MSCs) treatment with or without prednisone on these parameters has never been studied in dogs with CIE. These parameters were compared between healthy dogs (n = 12) and dogs with CIE, and before and 1, 3, 6, and 12 months after the treatment with MSCs alone (n = 9) or together with prednisone (n = 11). The relationship between the Canine Inflammatory Bowel Disease Activity Index (CIBDAI) and oxidative stress was evaluated. Albumin was the only parameter that significantly differed between dogs with CIE and healthy dogs (*p* = 0,037). Differences were observed only in albumin values after combined treatment with MSCs and prednisone. No differences were observed in MDA and GSH after treatment with MSCs with or without prednisone. Albumin could help stage canine CIE, as well as its prognosis, as has already been demonstrated, although it is essential to evaluate this parameter for its antioxidant capacity, and therefore it could be a good biomarker of oxidative stress in this pathology. However, the treatment with MSCs seems unable to modify any of the analyzed oxidative stress parameters.

## Introduction

Canine chronic inflammatory enteropathy (CIE) comprises a chronic idiopathic inflammation of the gastrointestinal mucosa tract alternating periods during which the patient is stable with flares of disease activity (Dandrieux 2016). According to whether the patient responds to dietary changes or immunosuppressant therapy the disease is called food-responsive enteropathy or immunosuppressant-responsive enteropathy, also known as IBD (inflammatory bowel disease). Until recently, a third category named antibiotic-responsive diarrhea (ARE) was recognized as one form of canine enteropathy. However, more recent works recommended avoiding the empirical antimicrobial treatment trial in the diagnostic work-up of canine CIE, due to the detrimental effects of the indiscriminate use of antibacterial drugs on the patient and the general population (Cerquetella et al. 2020). In addition, the low number of dogs responsive to antibiotics casts doubts on the true existence of an ARE group (Cerquetella et al. 2020; Jergens and Heilmann 2022).

Patients that do not respond to any of the treatments are included in the non-responsive enteropathies group (Dandrieux 2016; Isidori et al. 2022).

Oxidative stress has been demonstrated to play an important role in the pathogenesis of IBD in humans and dogs (Rezaie et al. 2007; Rubio et al. 2016a, b a, b; Segarra et al. 2016; Yuksel et al. 2017; Rubio et al. 2017). Oxidative stress occurs due to the excessive production of oxidative radicals or the lack of antioxidant molecules (Strober et al. 2007). In IBD, the excessive immune response that occurs secondary to chronic inflammation and tissue poor perfusion due to mucosal damage leads to the overproduction of reactive oxygen and nitrogen species (ROS/RNS). Structural modification and functional inhibition of cellular lipids, proteins, carbohydrates, and DNA associated with ROS/RNS-derived oxidative damage contribute to the development and progression of inflammation of the intestinal mucosa (Pravda 2005; Circu and Aw 2011).

Because of all these characteristics, certain antioxidants and oxidative compounds such as Trolox equivalent antioxidant capacity (TEAC), cupric reducing antioxidant capacity (CUPRAC), paraoxonase 1 (PON1) ferric reducing ability of plasma (FRAP) and total serum thiol concentrations have been studied to evaluate the antioxidant response and oxidative damage in dogs with IBD (Rubio et al. 2016a, b a, b; Segarra et al. 2016; Rubio et al. 2017).

Other oxidative stress parameters, studied mainly in tissues and biological fluids from humans, include malondialdehyde (MDA), reduced glutathione (GSH), and albumin (Anderson 1998; Sitar et al. 2013; Rubio et al. 2017).

MDA is produced during lipid peroxidation, a process involved in the pathogenesis of numerous inflammatory diseases and malignancies. It promotes intramolecular or intermolecular protein/DNA cross-linking, altering the biochemical properties of biomolecules, leading to different pathological states (Ayala et al. 2014). GSH is an endogenous antioxidant that acts against reactive nitrogen intermediates and has detoxifying effects against malignant endobiotics and xenobiotics. It is therefore a good indicator of cell functionality and viability and has been associated with multiple pathological processes (Denzoin Vulcano et al. 2013). Albumin is the most abundant protein in the body and is used as a marker of protein reserves and nutritional status, although it is also an important extracellular antioxidant molecule. Its antioxidant properties include the elimination of free radicals and the provision of the thiol group (Tabata et al. 2021).

In humans, these three parameters have been shown to be biomarkers of oxidative stress in Crohn's disease (CD) (Boehm et al. 2012; Szczeklik et al. 2018; Su et al. 2019). In veterinary patients, albumin has been studied in dogs with CIE from the point of view of nutritional status; however, recently, in multiple investigations, more importance has been given to the redox status of albumin as an aggravating factor in multiple pathologies, which could be important when creating new drugs (Tabata et al. 2021). Furthermore, in patients with CIE it has been described as an important marker of poor prognosis (Volkmann et al. 2017).

In veterinary medicine, the oxidative role of MDA has been studied in dogs with atopic dermatitis (Kapun et al. 2012), congestive heart failure (Nemec Svete et al. 2021), different types of cancer (Macotpet et al. 2013), dogs infected by Babesia (Crnogaj et al. 2010) and cats infected by coronavirus (Kayar et al. 2015). GSH has been studied in dogs and cats with hepatopathies (Center et al. 2002), cardiovascular diseases, numerous tumors (Viviano et al. 2009), and hemolytic and non-hemolytic anemia (Woolcock et al. 2020). However, they have never been investigated in patients with CIE.

Moreover, the safe administration and the anti-inflammatory capacity of allogeneic adipose-derived mesenchymal stem cells (MSCs) in the treatment of CIE in dogs has been described in previous research (Pérez-Merino et al. 2015; Cristóbal et al. 2021, 2022). Although the antioxidant properties of the MSCs, exerted either by decreasing the activity of oxidizing agents or promoting the antioxidant defenses, have been demonstrated in different diseases, such as gastrointestinal inflammation, and ischemic injuries (Eiro et al. 2022), the variation in oxidative stress after the application of this novel cell therapy in dogs with CIE remains unexplored.

Considering the above, the present study has two aims. The first is to evaluate the plasma concentration of the oxidative stress biomarkers MDA, GSH, and albumin in healthy dogs and dogs with CIE. The second aim is to investigate possible changes in these parameters in dogs with CIE treated with MSCs with or without concomitant prednisone.

## Materials and Methods

This research was developed at the Veterinary Teaching Hospital of the University of Extremadura. The Animal Care and Use Committee of the UEx and the Government of Extremadura approved this study (File No. 20160822, approved 22 August 2018). The owners of the animals included in the study were informed in detail and signed an informed consent.

### Groups

A control group of clinically healthy dogs over one year old, with no digestive signs or other pathologies and no treatments in the last year, attending the internal medicine clinic of the Veterinary Teaching Hospital of the University of Extremadura (VTH-UEx) for routine check-ups, was formed to compare oxidative stress biomarkers between dogs with CIE and healthy dogs. The absence of alterations in routine blood tests or abdominal ultrasound was also an inclusion criterion for this group.

A group of dogs with CIE was formed including dogs over one year of age, with digestive signs of more than three weeks of evolution that did not respond to diet, antibiotic, and immunosuppressant-based treatments previously established according to standard guidelines (Jergens and Heilmann 2022). Complete anamnesis, physical examination, complete blood count and blood biochemistry (including measurement of albumin, folic acid, cobalamin, and trypsin-like immunoreactivity [TLI]), urinalysis, coprological analysis with Giardia test and abdominal ultrasound were performed for the diagnosis of CIE. Those in which inflammation was confirmed by histopathological analysis of endoscopic biopsies were included. Dogs with other pathologies, sepsis, physical damage, and pregnant bitches were excluded.

The group of dogs with CIE was subdivided into two treatment groups to compare the effect of treatment on oxidative stress biomarkers in dogs with CIE:

- MSCs group: untreated dogs at least 21 days prior to MSCs administration.
- MSCs + Prednisone (P) group: dogs treated with prednisone (doses between 0.75 and 2 mg/kg) due to worsening symptomatology during the washout period of 21 days prior to MSCs infusion. After treatment, if the Canine Inflammatory Bowel Disease Activity Index (CIBDAI), described by Jergens and colleagues (Jergens et al. 2003), improved (with a decrease of more than 30% from the previous value), the prednisone dose was reduced. If not, the prednisone dose was not changed.

### Study design

Blood was collected from all dogs in both groups prior to any treatment and plasma was frozen at -85 °C to analyze and compare MDA, GSH, and albumin levels between the Control Group and CIE group. The resulting CIBDAI of the CIE dogs was related to the three oxidative stress biomarkers.

A single dose of adipose tissue-derived allogeneic MSCs was administered to patients with CIE from the MSCs and MSCs + P groups. Intravenously, 4 × 10^6^ cells/kg body weight diluted in physiological saline (100—250 ml depending on the weight of the animal) was infused over 30 min. Blood samples were collected to determine MDA, GSH, albumin levels, and CIBDAI scores were obtained at one (T1), three (T3), six (T6), and 12 (T12) months after MSCs administration (T0). In each group, the difference between all screening times before and after treatment was assessed by relating CIBDAI to oxidative stress biomarkers.

### MSCs culture

Subcutaneous adipose tissue was obtained from a female dog undergoing conventional spay surgery. MSCs were extracted from this tissue, which was digested with collagenase type V, washed, and filtered. MSCs were cultured and expanded in Dulbecco's Modified Eagle Medium (DMEM) with 10% fetal bovine serum (FBS) and penicillin/streptomycin at 37°C and 5% CO_2_. Using flow cytometry, following the guidelines of the International Society for Cell Therapy (Dominici et al. 2006), adherent cells were phenotypically characterized, in addition to differentiating in vitro to chondrogenic, osteogenic and adipogenic lineages. The expanded MSCs were cryopreserved until use, at which time they were thawed and resuspended in 50 ml of physiological saline.

### Determination of Oxidative Stress Biomarkers

MDA was estimated according to the spectrophotometric method of Ohkawa et al. adapted for microplate assays (Ohkawa et al. 1979). Plasma extracts were incubated at 95°C for 1 h in a mixture of 20% acetic acid (adjusted to pH 3.5 using NaOH), 8.1% SDS (with 0.05% butylated hydroxytoluene), and 0.8% solution of thiobarbituric acid. Then, a solution of n-butanol:pyridine (15:1, v/v) was added to the reaction mixture, shaken (5 min), and centrifuged (10,000 × g at 4°C for 5 min). The upper organic layer (pink-colored) was removed and read at 532 nm. MDA formation was expressed as micromoles of MDA equivalents per milligram of total protein using a calibration curve of 1,1,3,3-tetramethoxypropane (6.25–100 nmol/mL).

GSH was determined according to the fluorometric method described by Hissin and Hilf (Hissin and Hilf 1976). Samples were deproteinized by the addition of 50% cold trichloroacetic acid and then centrifuged (10,000 × g at 4°C for 10 min). Afterward, samples (50 µL) were incubated with 1 mg/mL of the fluorescent reagent o-phthalaldehyde in 0.1 M sodium phosphate (pH 8.0) containing 5 mM EDTA. The reaction mixture was incubated at 20°C for 45 min, followed by fluorimetric measurement (the excitation and emission wavelengths were set at 420 and 350 nm, respectively). The calibration curve of GSH (3.20–320 µmol/mL) as an external standard was used for quantification.

Albumin analysis was performed using an automatic blood biochemistry analyzer (Saturno 100 Vetcrony® Instruments, Rome, Italy) and a Spinreact® commercial laboratory kit (Spinreact SA, Girona, Spain).

### Statistical Analysis

Graphpad Prims software (version 8) was used to analyze the data. Concentrations of oxidative stress markers (MDA, GSH, and albumin) and CIBDAI index were compared between healthy dogs and CIE dogs. In addition, the values of these parameters were compared before and after treatment with MSCs at different times. A study of the normality of each component was performed using the Shapiro–Wilk test. The results were not normally distributed, so they were presented as the median and interquartile range (IQR).

Comparisons between the parameters of the control group and the CIE group were established using the Mann–Whitney U test. For analysis of changes over time, the Kruskal–Wallis test was performed. Dunn's test was used as a post hoc test. Spearman's test was used to study the correlations between MDA, GSH, and Albumin and the CIBDAI scores. The degree of significance was considered if *p* < 0.05.

## Results

### Study Population

The control group consisted of 12 healthy dogs of different breeds: Border Collie (n = 3), mixed breed (n = 3), Spanish Water Dog (n = 2), Jack Russel (n = 1), Samoyedo (n = 1), Poodle (n = 1), and English Setter (n = 1) of which five were females and seven were males. The median and range (minimum–maximum) age of the dogs was 2.75 (1.5—8.5) years, and the median and range (minimum–maximum) weight was 16.7 (2.60 to 21.5) kg.

The group of dogs with CIE included 20 dogs subdivided into 9 dogs in the MSCs group and 11 in the MSCs + P group. The breeds included were German Shepherd (n = 4), mixed-breed (n = 4), French Bulldog (n = 2), Yorkshire terrier (n = 2), Poodle (n = 1), Mastiff (n = 1), Greyhound (n = 1), Boxer (n = 1), Pomeranian (n = 1), Spanish Water Dog (n = 1), Husky (n = 1), Golden Retriever (n = 1). The median and range (minimum–maximum) age of the dogs was 4 (1—14) years, and the median and range (minimum–maximum) weight was 15.9 (2 to 26) kg. There were 13 males and 7 females. Dogs with CIE showed a median and range (minimum–maximum) of the CIBDAI value of 7.5 (5 – 14). According to this index, 8 dogs had severe disease (CIBDAI ≥ 9), 10 moderate disease (CIBDAI 6–8), and 2 mild disease (CIBDAI 4–5).

### Oxidative Stress Biomarkers in healthy dogs versus dogs with CIE

**Fig. 1:**
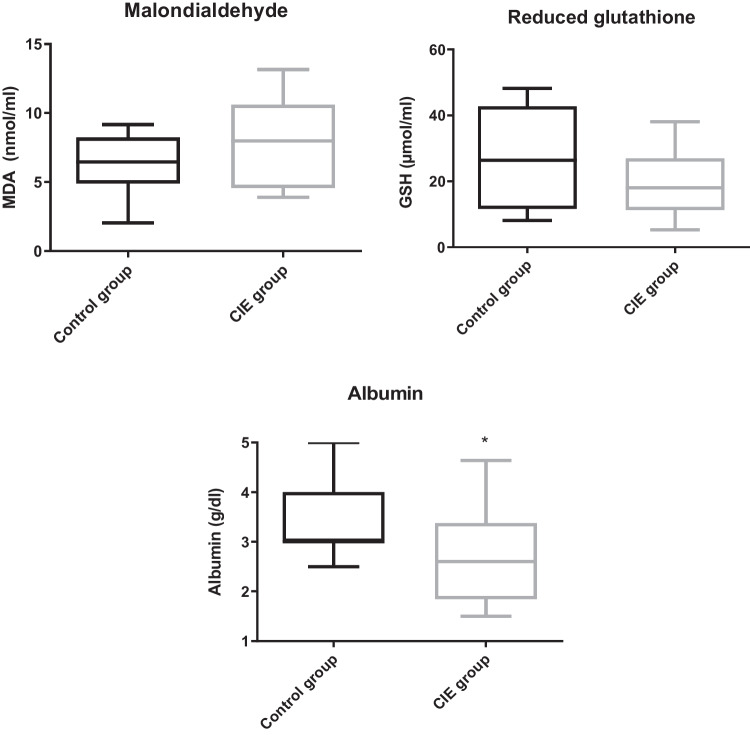
Comparison of malondialdehyde (MDA), reduced glutathione (GSH), and albumin between the control and chronic inflammatory enteropathy (CIE) groups. Asterisks indicate significant differences with the other group

Albumin was significantly lower in the CIE group before the treatment than in the control group (*p* = 0.037). No statistical differences were observed for the MDA and GSH between groups (*p* = 0.58 and *p* = 0.27, respectively). (Fig. 1 and Table 1).

**Table 1:** The median and interquartile range of MDA, GSH and albumin in control dogs and dogs with CIE plasma

	MDA	GSH	Albumin
CONTROL GROUP	6.46 (3.33)	26.39 (31.06)	3.00 (0.63)
CIE GROUP	7.98 (6.14)	18.00 (15.67)	2.60 (0.98)*

*Significant differences with the other group (*p* < 0.05)

### Changes in CIBDAI index and oxidative stress biomarkers after treatment with MSCs

**Fig. 2:**
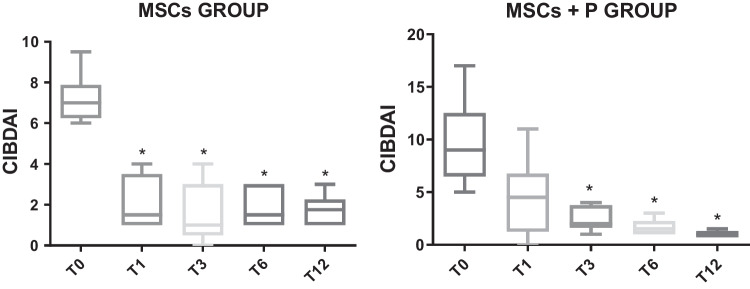
Canine Inflammatory Bowel Disease Activity Index (CIBDAI) in dogs with chronic inflammatory enteropathy (CIE) before treatment (T0) with mesenchymal stem cells (MSCs) or with MSCs and prednisone (MSCs + P) and at different review times: one (T1), three (T3), six (T6), and 12 months (T12). Asterisks indicate significant differences with T0

CIBDAI decrease was more pronounced at T1. It stabilized at T3 with a slight increase at 6 and 12 months. All post-treatment times present significant differences with respect to T0 (*p* = 0.005) (Fig. 2 and Table 2).

**Table 2:** The median and interquartile range of MDA, GSH, albumin and CIBDAI scores pretreatment (T0) and one (T1), three (T3), six (T6), and 12 months (T12) after treatment in dogs with CIE treated with mesenchymal stem cells (MSCs group) alone o combined with prednisone (MSCs + P group)

	MSCs group			MSCs + P group				
T0 T1 T3 T6 T12	MDA T12 4.15 (2.29) 3.35 (3.75) 2.54 (1.83) 6.11 (7.69) 2.80 (1.12)	GSH 28.06 (9.39) 27.15 (7.01) 26.20 (5.92) 25.99 (8.53) 25.28 (6.62)	ALBUMIN 3.20 (1.53) 3.10 (1.20) 3.20 (0.08) 3.40 (0.30) 3.65 (0.45)	CIBDAI 7.00 (1.63) 1.50 (2.50)** 1.00 (2.50)** 1.50 (2.00)** 1.75 (1.25)**	MDA 5.90 (7.17) 5.05 (4.16) 5.26 (4.31) 4.50 (5.09) 6.28 (4.76)	GSH 10.36 (3.02) 13.61 (6.02) 11.18 (3.15) 11.58 (3.94) 12.37 (2.83)	ALBUMIN 2.20 (1.15) 2.60 (0.65) 3.00 (1.10) 3.30 (1.13)** 3.30 (0.35)**	CIBDAI 9.00 (6.00) 4.50 (5.50) 2.00 (2.13)* 1.50 (1.25)* 1.00 (0)*

*Differences with T0 for that parameter (*p* < 0.05)

**Differences with T0 for that parameter (*p* < 0.01)

**Fig. 3:**
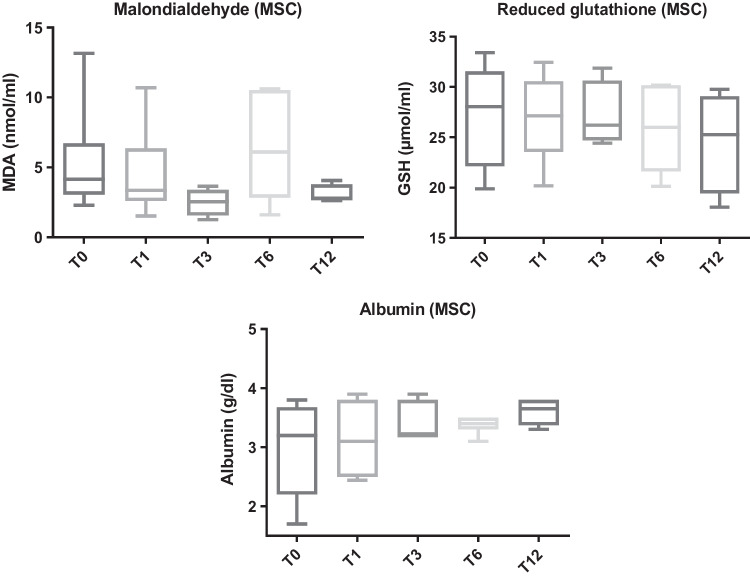
Malondialdehyde (MDA), reduced glutathione (GSH), and albumin in dogs with chronic inflammatory enteropathy (CIE) before treatment (T0) with mesenchymal stem cell (MSCs group) and at different review times: one (T1), three (T3), six (T6), and 12 months (T12)

No differences were observed in MDA, GSH, and albumin at any time point before and after the treatment (*p* = 0.30, *p* = 0.89, and *p* = 0.15, respectively) (Fig. 3 and Table 2).

### Changes in CIBDAI index and oxidative stress biomarkers after treatment with MSCs and prednisone

CIBDAI showed a progressive decrease after treatment with MSCs together with corticosteroids. CIBDAI values at T3, T6, and T12 differed significantly from T0 (*p* = 0.02) (Fig. 2 and Table 2).

**Fig. 4:**
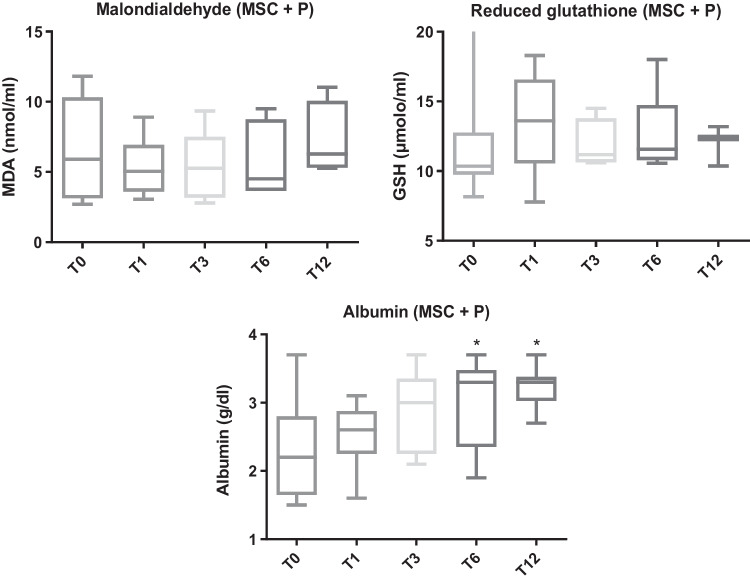
Malondialdehyde (MDA), reduced glutathione (GSH) and albumin in dogs with chronic inflammatory enteropathy (CIE) before combination treatment (T0) with mesenchymal stem cell and prednisone (MSCs + P group) and at different review times: one (T1), three (T3), six (T6), and 12 months (T12). Asterisks indicate significant differences with T0

No difference was found in MDA and GSH in dogs with CIE before and at any time point after the treatment with MSCs and prednisone (*p* = 0.84 and *p* = 0.47, respectively). Significant differences were observed in albumin between T0 and T6 (*p* = 0.008) and between T0 and T12 (*p* = 0.006) (Fig. 4 and Table 2).

### Correlation study

A significant positive correlation was found between the changes in the CIBDAI scores and the MDA (ρ = 0.62; *p* = 0.05) and GSH (ρ = 0.43; *p* = 0.05) values after the treatment in the MSCs + P group. No other significant correlation was observed.

## Discussion

Due to the strong implication of oxidative stress in the pathophysiology of CIE, different oxidative and antioxidant biomarkers are currently being increasingly investigated. In the present study, an analysis of biochemical parameters related to oxidative stress in dogs with CIE was carried out compared with healthy animals. In turn, changes in these biomarkers after administration of MSCs alone or in combination with prednisone were evaluated.

In dogs with CIE, antioxidant serum biomarkers TEAC, CUPRAC, and PON1, have been shown to be decreased with respect to healthy dogs, but not FRAP (Rubio et al. 2016a, b, 2017; Segarra et al. 2016). In a recent study, dogs exhibited an increase in determinable reactive oxygen metabolites (dROMs), and Oxidative Stress index (OSi), whereas no difference was found in Serum Antioxidant Capacity (SAC), compared to healthy dogs (Candellone et al. 2022).

Nevertheless, there was no data in the veterinary literature relating to MDA in canine CIE. In humans, the results of the changes in MDA during Crohn's disease are not consistent. Some studies described an increase in MDA values in CD patients, thus supporting an increase in free radicals in these patients and demonstrating an important role of oxidative stress in this disease (Alzoghaibi et al. 2007; Boehm et al. 2012; Achitei et al. 2013) and even the association of the increase of MDA in saliva and serum with the Crohn’s disease activity index (Szczeklik et al. 2018). However, as happened in the present study, MDA has been reported to be unchanged in the colon of patients with ulcerative colitis or Crohn’s disease (Bhaskar et al. 1995; Koch et al. 2000, 2002; Tüzün et al. 2002). The differences in these results have been attributed to the different origins of the samples examined (human plasma, breath alkanes, or mucosal biopsies) and to the different biochemical techniques utilized to estimate free radical production (Karp and Koch 2006).

In dogs and cats, higher MDA values were found in dogs with age-related cataracts (Madany 2016), cancer-bearing dogs (Macotpet et al. 2013), Ehrlichia canis (Çiftci et al. 2021) and parvo-infected dogs (Gaykwad et al. 2018), and dogs with atopic dermatitis (Kapun et al. 2012), but the present study has failed to show that increase in dogs with CIE.

GSH decrease has been reported in ill dogs affected by several diseases including a small number of them affected by gastrointestinal diseases compared to healthy animals (Viviano et al. 2009). In our study, however, that difference between dogs with CIE and healthy dogs was not observed. In humans, a study showed a decrease in the GSH in both the healthy and inflamed ileum of patients with CD compared to the ileum of healthy control patients (Iantomasi et al. 1994) and others in the colon (Holmes et al. 1998; Sido et al. 1998; Miralles-Barrachina et al. 1999). However, a study measuring GSH in the saliva of patients with CD has shown that GSH concentrations are decreased, but only in active CD (Szczeklik et al. 2018) and another demonstrated that there were no differences in colonic total antioxidant capacity comparing control colon to inactive and active ulcerative colitis in spite of the decreased tissue levels of GSH. This last study suggested that the depletion of glutathione in ulcerative colitis may be a specific disorder rather than a secondary defect attributable to global oxidative stress. It remains unclear whether glutathione depletion is related to increased consumption or to malnutrition that alters glutathione biosynthesis (Koch et al. 2000; Karp and Koch 2006).

Our results showed that the albumin value was decreased in CIE dogs relative to the control group as observed in human patients together with the depletion of other antioxidant markers such as FRAP, GSH, bilirubin, and uric acid (Szczeklik K et al. 2018; Su et al. 2019). This alteration has been consistently reported in many studies on canine CIE, and it has been considered a parameter of poor prognosis (Craven et al. 2004; Allenspach et al. 2007; Cristóbal et al. 2021). However, those studies do not approach the study of albumin from the point of view of its antioxidant qualities. Serum albumin is involved in redox homeostasis in the circulation. The most abundant thiol in plasma is the Cys34 residue, which is found in albumin. It exerts antioxidant activity, prevents systemic oxidative stress, and eliminates ROS and RNA. Furthermore, it has been shown that, as albumin molecules are oxidized by free radicals, the antioxidant capacities of this protein decrease. Therefore, researchers are currently demonstrating that oxidized albumin is a good biomarker of oxidative stress, as well as being an aggravating factor in multiple pathologies (Tabata et al. 2021).

Moreover, several features of the MSC therapy have been demonstrated in relation to oxidative stress, including direct antioxidant effects, such as the scavenging of free radicals and the donation of healthy mitochondria to damaged cells, and indirect effects, such as the enhancement of antioxidant defenses in other cells of the body and the alteration of cellular bioenergetics. In addition, the immunosuppressive effects of MSCs prevent the generation of ROS. All these characteristics lead to a decrease in oxidative stress, generating beneficial effects against multiple pathologies (Inan et al. 2017; Jung et al. 2020; Stavely and Nurgali 2020; He et al. 2021).

It has been shown that MSCs could repair damaged brain tissue and decrease oxidative stress levels in humans by inhibiting the production of ROS/RNS (Calió et al. 2014). Another study showed the therapeutic effect of MSCs on damaged small intestine tissue from humans with intestinal ischemia/reperfusion was associated with increased antioxidant capacity and decreased oxidative stress, as indicated by a lower level of MDA and increased activities of superoxide dismutase (SOD), catalase (CAT), and glutathione peroxidase (Gpx) (Inan et al. 2017). Similar findings were reported in mice in which treatment with MSCs was shown to decrease oxidative stress by reducing ROS production, decreasing the MDA level, and increasing the activity of antioxidant enzymes SOD, CAT, and Gpx (Jung et al. 2020).

Furthermore, other treatments seemed able to modify the biomarkers in dogs such as N-acetylcysteine treatment that proved to improve glutation S-transferase activity and decrease MDA concentrations in parvo-infected dogs (Gaykwad et al. 2018). However, in the present study, only albumin concentration improved but no changes in MDA and GSH levels were observed after the application of MSCs alone or in combination with prednisone in dogs with CIE.

One of the main limitations of this study is the low number of patients that we were able to include in each of the groups studied. The variability that may exist between the different breeds and sizes of the dogs included in the study is another limitation, as it may influence the values obtained for oxidative stress biomarkers. In addition, the limited literature on the determination of oxidative stress biomarkers in canine species makes it difficult to interpret the results obtained in our study.

In conclusion, when investigating the three biomarkers of oxidative stress in dogs with CIE, only differences in albumin values were observed, with no differences in MDA and GSH parameters. The treatment with MSCs, either alone or in combination with corticosteroids, was shown to be effective in CIE; however, only the albumin value varied (increasing) after the MSCs treatment, whereas the rest of the oxidative stress parameters analyzed were not significantly modified by the treatment. It would be of interest to consider albumin as a biomarker of oxidative stress, as well as a nutritional marker, due to its antioxidant qualities.

## Data Availability

The data presented in this study are available from the corresponding author upon reasonable request.
